# Analysis of the infra-acetabular corridor: sex-specific differences in the secure area and insertion angle in infra-acetabular screw placement

**DOI:** 10.20407/fmj.2019-014

**Published:** 2020-02-11

**Authors:** Masahiro Yoshida, Koji Sato, Tomohiro Ando, Masatoshi Haruta, Hiroaki Iwase

**Affiliations:** Department of Orthopaedic Surgery, Nagoya Daini Red Cross Hospital, Nagoya, Aichi, Japan

**Keywords:** Infra-acetabular corridor, Infra-acetabular screw, Corridor length, Corridor angle, Acetabular fracture

## Abstract

**Objectives:**

A majority of older adult acetabular fracture patients have a fracture of the anterior component, and repair of the acetabular anterior component with infra-acetabular screw (IAS) fixation is crucial. The aim of this study was to clarify the sex-specific differences in the secure infra-acetabular corridor for safe IAS placement.

**Methods:**

Three-dimensional pelvic computed tomography (CT) images of 50 males and 50 females with an average age of 77.5 years were analyzed. The secure insertion path of IAS was simulated on the ZedHip system (Lexi Co., Ltd., Japan), and length, angle, and diameters of the infra-acetabular corridor were measured.

**Results:**

The lengths of the corridors were 99.0±4.6 mm in males and 91.5±5.3 mm in females (p<0.01). The angle of the corridor to Y axis in the axial plane on the functional pelvic plane (FPP) was 5.1±4.9° in males and 8.6±5.3° in females (p<0.01). However, in 32% of the cases it was deemed that a IAS could not be inserted because the diameters of the corridor were too narrow to insert the screw.

**Conclusions:**

On simulation, the corridor length was shorter and corridor angle was larger in females. In one third of cases the infra-acetabular corridor simulation showed it was impossible to insert the IAS, so it is crucial to scrutinize the infra-acetabular corridor on CT images during preoperative planning for IAS insertion in acetabular fractures.

## Introduction

Pelvic fractures are some of the most challenging fractures for orthopedic trauma surgeons.^1–3^ Anatomic reduction and rigid fracture fixation enable early joint movement and decrease the incidence of postoperative complications, such as posttraumatic osteoarthritis, thrombosis, muscle atrophy, and pneumonia.^4,5^

Pelvic insufficiency fractures have been increasing over the past few decades with the aging population, and 64% of acetabular fractures include a fracture of the anterior wall and/or the anterior column.^6^

**Figure 1:**
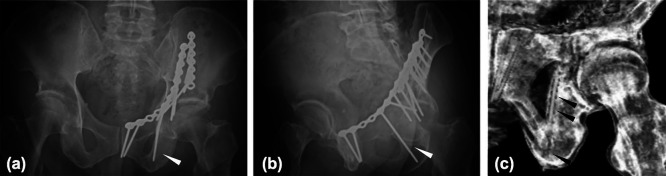
X-ray of the infra-acetabular screw. (a) AP view, (b) obturator-oblique view and (c) 3D-CT of the postoperative X-ray of the infra-acetabular screw (IAS) used in the case of an acetabular fracture. Arrowheads: IAS

The infra-acetabular corridor is a narrow bony path from the acetabulum to the ischial tubercle, and inserting a screw in this path (called an infra-acetabular screw (IAS)) (Figure 1) requires skill.^7–9^ The IAS, in addition to the posterior column screw, doubles the static fixation strength by constructing the “periacetabular fixation frame”, rather than the posterior column alone, in the fixation of acetabular fractures.^10^

However, there are individual differences in infra-acetabular corridor size and the axis of the IAS path.^11^ Such considerations have not been made so far in Japanese pelvises, which might have racial differences from those of overseas reports. We conducted a study targeting Japanese pelvic bone morphology by analyzing the data of 100 pelvis computed tomography (CT) scans. The aim of this study was to clarify the Japanese sex-specific differences in the secure corridor size and angle in older adult pelvises to determine IAS placement in acetabular fractures.

## Methods

This study was conducted with permission of the ethics committee of Nagoya Daini Red Cross Hospital.

CT DICOM data of 100 pelvises were obtained from 50 males and 50 females over the age of 60 years old who had obtained a pelvic CT for hip disorders between 2016 and 2018. The cases included 63 patients with hip fracture (HF), 28 with hip osteoarthritis (OA), and 9 with osteonecrosis (ON). The average age was 77.5 years old, and there was no significant difference in age distribution between males and females (Table 1).

**Figure 2:**
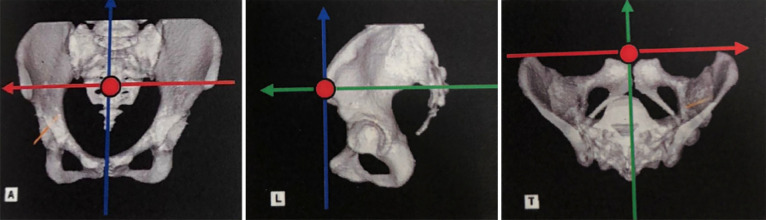
Axis of functional pelvic plane. Red circle: Origin point=midpoint of the anterior superior iliac spine (ASIS). Red arrow: X axis=ASIS digitize point line. Blue arrow: Z axis=parallel to bed and perpendicular to X axis. Green arrow: Y axis=perpendicular to X and Z axis

**Figure 3:**
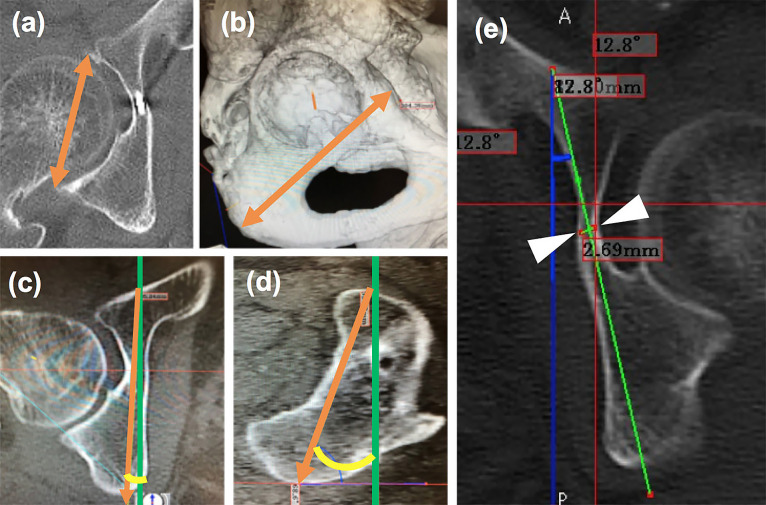
Parameters measured using the ZedHip system. (a) acetabular size. (b) corridor length. Angle between the arrow and Y axis (green line) is the corridor angle in the axial plane (c) and in the sagittal plane (d). Corridor diameter is the distance between the white arrow heads (e).

(1) Acetabular size, (2) corridor length, (3) corridor angle, and (4) minimum corridor diameter were measured on the unaffected side by simulating a secure insertion path of the IAS in the ZedHip system (Lexi Co., Ltd., Japan). All measurements were performed based on the functional pelvic plane^12^ using the origin, X axis, Y axis, and Z axis (Figure 2).

(1)  Acetabular size was defined as the maximum distance from the acetabular anterior wall to the acetabular posterior wall (Figure 3a).
(2)  Corridor length was measured by simulating the maximum length of the IAS inserting from the “tear drop”, as described by Culemann^7^ (Figure 3b).
(3)  Corridor angle was measured by simulating the most suitable angle in inserting the IAS, and the angle with the Y axis was measured in the axial plane and sagittal planes (Figures 3c, d).
(4)  Corridor diameter was the minimum diameter of the IAS path in the infra-acetabular corridor (Figure 3e).

### Statistical analysis:

For statistical analysis, Microsoft Excel (Microsoft Inc. Redmond, WA, USA) and the software program EZR^13^ were used. Data are presented as mean±SD. The continuous variable was analyzed using Student’s t-test, and the factor variable was analyzed using Fisher’s exact test. P values smaller than 0.05 were considered statistically significant.

## Results

Acetabular size was 52.3±2.5 mm in males and 48.3±2.8 mm in females, and the value in males was significantly larger (p<0.01). Corridor length was also significantly longer in males (99.0±4.6 mm) compared with females (91.5±5.3 mm) (p<0.01). The corridor angle in the axial plane was 5.1±4.9° in males and 8.6±5.3° in females, which was significantly larger in females (p<0.01). The corridor angle in the sagittal plane was not significantly different between males (34.8±8.4°) and females (33.2±9.3°). The minimum corridor diameter was 3.7±1.3 mm in males and 3.4±1.1 mm in females (n.s.). The corridor minimum diameter of 26 males (52%) and 29 females (58%) was less than 3.5 mm, and in 15 males and 17 females (32% of all cases) the IAS was unable to be inserted because the straight trajectory for the IAS in the corridor was unable to be placed on simulation due to a thin and curved corridor (Table 2).

## Discussion

The incidence of acetabular fractures in older adults has increased worldwide, and acetabular fracture cases in older adults are more likely to involve the anterior column than those in younger individuals.^14^ To achieve a successful functional outcome in acetabular fractures, the IAS provides rigid fracture fixation.^7,11,15^ Arlt et al. evaluated the data from 124 pelvic CTs and reported that corridor volume was significantly larger in males compared with females, and 97% of males and 91% of females have enough corridor size for IAS placement.^16^ They also mentioned that hip dysplasia has no correlation with parameters of the infra-acetabular corridor.^16^ Our data include OA and ON cases, but the parameters of the unaffected side had the same trend compared with HF cases. Gras et al. analyzed the data of 523 pelvic CT scans and reported that corridor diameter was 5 mm or more in 93% of cases, and average corridor length was significantly longer in males (106.4 mm on average) than in females (96.2 mm on average).^11^ They also indicated that the corridor angle in the axial plane was significantly larger in females: –0.3° in males and 4.3° in females.^11^ As shown by our results, the corridor angle in the axial plane was also significantly larger in females (Table 2).

**Figure 4:**
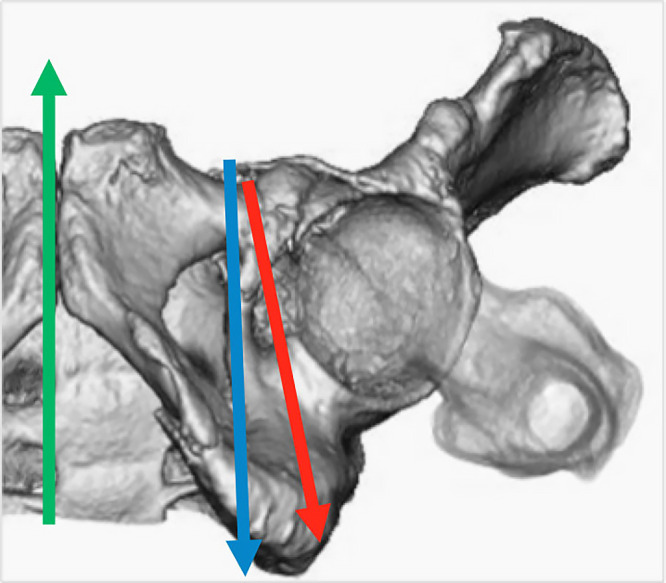
Appropriate IAS direction in the axial plane for female (red arrow) and for male (blue arrow). Green arrow: Y axis.

Considering these facts, the trajectory of the IAS in females was more in the lateral direction. However, the IAS should be placed nearly parallel to the Y axis in males (Figure 4). In addition, seven cases (14%) in males required a slight medial tilt in the axial plane.

Furthermore, although reports from abroad have revealed that more than 90% of cases have enough corridor area for IAS placement,^15,16^ in 32% of our cases it was judged that the IAS was unable to be inserted on simulation. Corridor length was also shorter in our cases than in reports from abroad,^15,16^ so racial difference may contribute to the difference in pelvic bone morphology.

Although the sex differences in pelvic size are known from previous reports,^17,18^ we have not corrected the length parameters by physical sizes such as height or weight between the sexes in this study. However, the angle of the corridor is not be influenced by the size of pelvis. IAS length is easily measured by fluoroscopy or depth gauge during the operation, but the insertion angle depends mostly on the skill of the surgeon, and it is important to recognize the approximate angle before the operation.

To our knowledge, no reports have investigated the sex-specific differences in the infra-acetabular corridor angle in the pelvic CT scans of Japanese patients. Because acetabular fractures in older adults are also increasing in this country,^19^ orthopedic trauma surgeons are increasingly encountering cases that require surgery. Considering the narrow corridor and its angle, preoperative CT scan analysis for each individual is necessary, and the visualization of the corridor area supports the surgeon when assessing the optimal IAS path in treating acetabular fractures.

## Figures and Tables

**Table1:** Characteristics of pelvis specimens

	Total (n=100)	Male (n=50)	Female (n=50)	Significance
Age (years)	77.5±9.6 (60–93)	77.0±10.7 (61–92)	77.8±8.4 (60–93)	NS
Diagnosis of affected side				
HF	63	33	30	NS
OA	28	11	17	
ON	9	6	3	

NS=not significant, HF=hip fracture, OA=osteoarthritis, ON=osteonecrosis

**Table2:** Parameters of infraacetublum corridor

	Total (n=100)	Male (n=50)	Female (n=50)	Significance
Acetabular size (mm)	50.3±3.3	52.3±2.5	48.3±2.8	p<0.01
Corridor length (mm)	95.3±6.2	99.0±4.6	91.5±5.3	p<0.01
Corridor angle (°)				
– axial plane	6.8±5.4	5.1±4.9	8.6±5.3	p<0.01
– sagittal plane	34.0±8.9	34.8±8.4	33.2±9.3	NS
Minimum corridor diameter (mm)	3.5±1.2	3.7±1.3	3.4±1.1	NS
– Cases under 3.5 mm	55	26	29	NS
– Cases unable to place IAS	32	15	17	NS
